# Relationship between lymphocytes and idiopathic macular hole

**DOI:** 10.1186/s12886-024-03424-7

**Published:** 2024-04-23

**Authors:** Ying Gao, Yun Tang, Ting Yu, Ying Ding, Yilu Chen, Wei Ye, Changlin Zhao, Rongxin Lu

**Affiliations:** 1 Sir Run Run Hospital, Nanjing Medical University, Nanjing, Jiangsu Province China; 2 Department of Ophthalmology, Affiliated Jinling Hospital, Medical School, Nanjing University, Nanjing, Jiangsu Province China; 3 Department of Thoracic Surgery, The First Affiliated Hospital With Nanjing Medical University, Nanjing, Jiangsu Province China; 4 Medical School, Nanjing University, Nanjing, Jiangsu Province China; 5 The Affiliated Eye Hospital, Nanjing Medical University, Nanjing, Jiangsu Province China

**Keywords:** Idiopathic macular hole, Lymphocyte, Immunoinflammatory marker, Closure index

## Abstract

**Background:**

An idiopathic macular hole (IMH) is a full-thickness anatomic defect extending from the internal limiting membrane to the photoreceptor layer of the macula without any known cause. Recently, clinical laboratory markers of systemic inflammatory status derived from complete blood counts have been evaluated in ocular diseases. This study aimed to explore whether they could predict the development and progression of IMHs.

**Methods:**

A retrospective review of 36 patients with IMH and 36 sex-and-age-matched patients with cataracts was conducted. We collected complete blood counts of all participating individuals and calculated systemic immunoinflammatory indicators. The maximum base diameter of the IMH (BD), minimum diameter of the IMH (MIN), height of the IMH (H), area of the intraretinal cyst (IRC), and curve lengths of the detached photoreceptor arms were measured on optical coherence tomography (OCT) images. We used these values to calculate the macular hole index (MHI), tractional hole index (THI), diameter hole index (DHI), hole form factor (HFF), and macular hole closure index (MHCI). We performed a receiver operating characteristic (ROC) curve analysis of 30 patients with IMH who were followed up 1 month after surgery.

**Results:**

Lymphocyte counts were significantly higher in the IMH group. No other significant differences were observed between the IMH and control groups. Lymphocyte counts in the IMH group were significantly negatively correlated with MIN and BD and were significantly positively correlated with MHI, THI, and MHCI. However, lymphocyte counts were not significantly correlated with H, IRC, DHI, and HFF. In the ROC analysis, BD, MIN, MHI, THI, and MHCI were significant predictors of anatomical outcomes. According to the cut-off points of the ROC analysis, lymphocyte counts were compared between the above-cut-off and below-cut-off groups. Lymphocyte counts were significantly higher in the MIN ≤ 499.61 μm, MHI ≥ 0.47, THI ≥ 1.2, and MHCI ≥ 0.81 groups. There were no significant differences between the above-cut-off and below-cut-off BD groups.

**Conclusions:**

Although inflammation may not be an initiating factor, it may be involved in IMH formation. Lymphocytes may play a relatively important role in tissue repair during the developmental and postoperative recovery phases of IMH.

## Background

A macular hole (MH) is a full-thickness anatomic defect that extends from the internal limiting membrane (ILM) to the photoreceptor layer of the macula [1]. Gass described MH in terms of four clinical stages in 1995: stage I is a central yellow spot or yellow ring with loss of foveolar depression, but no vitreofoveal separation; stage II is a full-thickness hole < 400 μm in diameter; stage III is a full-thickness hole ≥ 400 μm in diameter with persistent hyaloid attachment; and stage IV is a full-thickness hole ≥ 400 μm with complete posterior vitreous detachment [2]. Many causes of macular holes have been identified, including trauma, uveitis, and high myopia [3]. However, systemic or ocular causes of idiopathic macular holes (IMHs) remain elusive [4]. IMHs cause severe impairment of central vision predominantly in women older than 60 years [5].

Although the exact pathogenesis of IMHs is unclear, the literature provides several explanations for the etiology of the disease: (1) anterior–posterior traction by the posterior vitreous cortex on the macular fovea has been extensively accepted as a possible cause [6, 7]; (2) ILM contains collagen fibrils, proteoglycans, basement membranes and plasma membrane of Müller cells, and the proliferation and contraction of the ILM may be one of the reasons for MH enlargement [8]; and (3) Tornambe proposed the hydration theory, in which a break in the inner retinal layer leads to the accumulation of vitreous fluid in the middle and outer retinal tissues [9]. Based on these theories, vitrectomy with peeling of the ILM combined with gas or silicone oil tamponade has become a common surgical method for the treatment of IMHs.

However, there are different views on treatment options for IMHs. Tornambe [9] suggested that not all patients with MH should undergo surgery with ILM removal. If optical coherence tomography (OCT) shows only direct traction of the posterior hyaloid on the inner retinal defect, simply removing the posterior hyaloid should suffice for the permanent closure of the MH. Eckardt et al. [10] found evidence of necrosis or degeneration in Müller cells of the inner boundary membrane that was removed surgically. Hayashi [11] found mild inflammation on fluorescein angiography in a recurrent MH. After initiation of topical steroid treatment, inflammation was reduced and the MH was subsequently closed. Previous studies on eye inflammations have used IMH patients as controls because they were considered free of inflammation; however, Li and Wang [4, 12] found that after IMH onset, there were cytokines, such as IL-17, IL-14, and IL-13, in the aqueous humor. Therefore, several researchers have reported medical therapies for MH, including topical steroids [13, 14], nonsteroidal anti-inflammatory drugs [15, 16], and topical anhydrase inhibitors [17–19]. In addition, these drugs have been combined with intravitreal injections of steroids or bevacizumab [20].

Clinical laboratory markers of systemic immunoinflammatory status derived from complete blood counts include lymphocyte count, leukocyte count, neutrophil count, monocyte count, eosinophil count, basophil count, platelet count, neutrophil-to-lymphocyte ratio (NLR), monocyte-to-lymphocyte ratio (MLR), platelet-to-lymphocyte ratio (PLR), and systemic immune-inflammation index (SII, platelet × neutrophil/lymphocyte). Furthermore, some of these markers have been evaluated in ocular diseases, such as central retinal artery occlusion [21], anterior ischemic optic neuropathy [22], retinopathy of prematurity [23], and diabetic macular edema [24].

However, no studies have investigated the relationship between these immunoinflammatory markers and IMH. Therefore, this study aimed to explore whether they are involved in the development and progression of IMH.

## Methods

This retrospective study was performed at the Ophthalmology Department of the Affiliated Jinling Hospital, Medical School of Nanjing University, from January 2013 to January 2022.

OCT is currently used for detailed assessment of IMHs and regarded as the diagnostic gold standard [25]. This study included 36 patients with IMH (IMH group) and 36 sex-and-age-matched patients with cataracts (control group). According to Gass staging, the IMH group was classified using OCT into three categories: stage II (11 patients), stage III (13 patients), and stage IV (12 patients). The study was conducted in accordance with the Declaration of Helsinki and informed consent was obtained from each participant. Ethics committee approval was obtained from the ethics committee of the Affiliated Jinling Hospital, Medical School of Nanjing University, approval number 2023DZKY-008–01.

### Inclusion and exclusion criteria

All patients with IMH underwent pars plana vitrectomy (23G), with ILM peeling and tamponing agents, performed by two surgeons with at least 3 years of experience in MH surgery. The tamponing agents used included disinfect air, 12–14% perfluoropropane (C3F8), or 20% sulfur hexafluoride (SF6). Some patients underwent simultaneous cataract surgery. Patients with other ocular diseases (other than refractive errors and cataracts) were excluded. Patients with a history of ocular or general surgery were also excluded. Additional exclusion criteria included systemic diseases (other than hypertension), including diabetes mellitus, systemic infectious disease, cancer, anemia, trauma, acute coronary syndrome, cerebral disease, thyroid-related disease, nephropathy, pregnancy, and allergic disease. Patients who received anti-inflammatory therapy were also excluded.

### Examinations

All patients underwent a complete ophthalmological examination, including best-corrected visual acuity, slit lamp examination, intraocular pressure check, pupil-dilated fundus examination, OCT, and routine blood tests. Preoperative and postoperative OCT images were obtained using OCT fundus photography (RTVue100-2; Optovue, Fremont, CA, USA). Venous blood samples were collected from the antecubital veins after overnight fasting. Complete blood counts were tested using an automatic hematology analyzer (Sysmex XE-5000; Kobe, Japan), including lymphocyte, leukocyte, neutrophil, monocyte, eosinophil, basophil, and platelet counts. The NLR (neutrophil/lymphocyte), MLR (monocyte/lymphocyte), PLR (platelet/lymphocyte), and SII (platelet × neutrophil/lymphocyte) were calculated.

### Measurements of OCT parameters

**Fig. 1:**
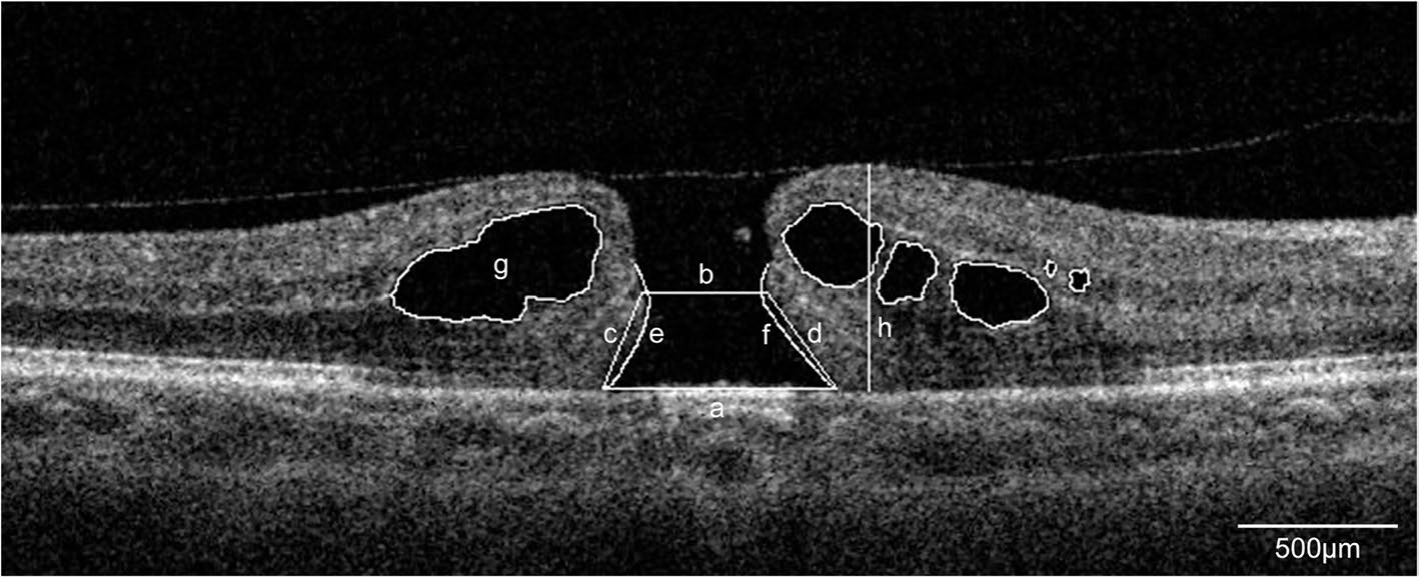
IMH parameters measured using OCT. **a** maximum base diameter; **b** minimum diameter; **c**, **d** arm length; **e**, **f** curvilinear distance between the break of the outer membrane and the starting point of photoreceptor detachment; **g** area of the intraretinal cyst; **h** height. MHI = h/a; THI = h/b; DHI = b/a; HFF = (c + d)/a; MHCI = (e + f)/a

Preoperative and postoperative OCT images were analyzed by three observers using Image-J (v1.53f51). The detection figures are shown in Fig. 1. The OCT parameters included the maximum base diameter of the MH at the level of the retinal pigment epithelium (BD), minimum diameter of the MH (MIN), height from the retinal pigment epithelium to the top of the MH (H), area of the intraretinal cyst (IRC), and curve lengths of the detached photoreceptor arms. Afterward, we calculated the macular hole index (MHI), tractional hole index (THI), diameter hole index (DHI), hole form factor (HFF), and MH closure index (MHCI) [26]. The derived indices were calculated as follows: Macular Hole Index (MHI) = Height/Maximum basal diameter;Tractional Hole Index (THI) = Height/Minimum hole diameter; Diameter Hole Index (DHI) = Minimum diameter/Maximum basal diameter; Hole Form Factor (HFF) = Nasal arm length + Temporal arm length/Maximum basal diameter; MH closure index (MHCI) = Curve length of the detached photoreceptor nasal arm + Temporal arm / Maximum basal diameter.

### Anatomical outcomes

In our study, based on the postoperative OCT images, the patients who completed the follow-up were divided into two groups based on the types of anatomical outcomes, as defined by Kang et al. [27]. We had type 1 closure, in which MH was closed without foveal defect of the neurosensory retina, and type 2 closure, in which MH still had a foveal defect of the neurosensory retina, although the entire edge of the MH was connected to the retinal pigment epithelium and the cuff was flattened.

### Statistical methods

SPSS software (version 21.0; IBM, Armonk, NY, USA) was used for the statistical analysis. Continuous data were summarized based on their distribution using either mean ± SD or median with the interquartile range (IQR). The Student’s t-test was used for normally distributed data within or between groups, and the Mann–Whitney U test was used for non-normally distributed data. The Pearson’s chi-square test was used to analyze categorical data. Pearson’s correlation analysis was used to analyze the relationship between lymphocyte count and normally distributed OCT parameters in the IMH group. Spearman’s correlation analysis was used to analyze the relationship between lymphocyte count and non-normally distributed OCT parameters in the IMH group. Receiver operating characteristic (ROC) curve analysis was used to determine the cut-off points for OCT parameters between type 1 and type 2 closures. According to the cut-off points, followed-up patients in the IMH group were divided into two groups, and lymphocyte counts were compared between groups using the Mann–Whitney U test. The results were evaluated within a 95% confidence interval, and statistical significance was set at *p* < 0.05.

## Results

A total of 72 participants were included in this study: 36 in the IMH group and 36 in the control group. The median (IQR) ages of the IMH and control groups were 63.00 (IQR 61.00–65.00) and 65.00 (IQR 62.00–69.00) years, respectively (*p* = 0.079). The study included 15 men and 57 women. The sex distribution was similar between the two groups (*p* = 0.772). The clinical characteristics and complete blood counts were shown in Table 1. The lymphocyte levels were significantly higher in the IMH group than in the control group (*p* = 0.029). There were no significant differences in other data between the two groups.

**Table 1:** Clinical characteristics and complete blood count

Parameters	IMH group *n* = 36	Control group *n* = 36	*p*-value
**Age**	63.00 (61.00–65.00)^d^	65.00 (62.00–69.00)^d^	0.079^a^
**Gender (M/F)**	7/29	8/28	0.772^b^
**Hypertension**	8	12	0.293^b^
**Lymphocyte(10**^**9**^**/L)**	1.91 ± 0.45^e^	1.71 ± 0.52^e^	0.029^c,^*
**Leukocyte(10**^**9**^**/L)**	5.43 (4.48–6.05)^d^	4.80 (4.46–5.80)^d^	0.554^a^
**Neutrophil(10**^**9**^**/L)**	2.77 (2.36–3.60)^d^	2.75 (2.15–3.35)^d^	0.774^a^
**Monocyte(10**^**9**^**/L)**	0.32 ± 0.07^e^	0.33 ± 0.10^e^	0.490^c^
**Eosinophil(10**^**9**^**/L)**	0.12 (0.06–0.19)^d^	0.12 (0.08–0.16)^d^	0.550^a^
**Basophil(10**^**9**^**/L)**	0.02 (0.01–0.03)^d^	0.01 (0.01–0.02)^d^	0.300^a^
**Platelet(10**^**9**^**/L)**	204.39 ± 48.20^e^	209.25 ± 47.52^e^	0.668^c^
**NLR**	1.39 (1.21–1.83)^d^	1.62 (1.34–2.06)^d^	0.105^a^
**MLR**	0.17 (0.13–0.20)^d^	0.19 (0.16–0.24)^d^	0.082^a^
**PLR**	108.00 (78.54–127.30)^d^	131.48 (103.11–155.63)^d^	0.081^a^
**SII**	297.5 (195.77–358.63)^d^	362.25 (280.33–430.27)^d^	0.056^a^
**FBG (mmol/L)**	4.74 ± 0.42^e^ (*n* = 32)	4.96 ± 0.45^e^ (*n* = 31)	0.051^c^
**FIB (g/L)**	2.63 ± 0.31^e^ (*n* = 25)	2.73 ± 0.43^e^ (*n* = 27)	0.348^c^
**Hb (g/L)**	135.00 (125.00–143.50)^d^	135.00 (126.00–141.00)^d^	0.735^a^

*NLR* Neutrophil-to-lymphocyte ratio, *MLR* Monocyte-to-lymphocyte ratio, *PLR* Platelet-to-lymphocyte ratio, *SII* Systemic immune-inflammation index, *FBG* Fasting blood glucose, *FIB* Fibrinogen, *Hb* Hemoglobin

^*^Statistically significant

^a^Mann-Whitney U test

^b^χ^2^ test

^c^Student’s t test

^d^non-normally distributed data were represented in medians (IQR)

^e^normally distributed data were represented in mean ± SD

### Lymphocyte count and OCT parameters

**Fig. 2:**
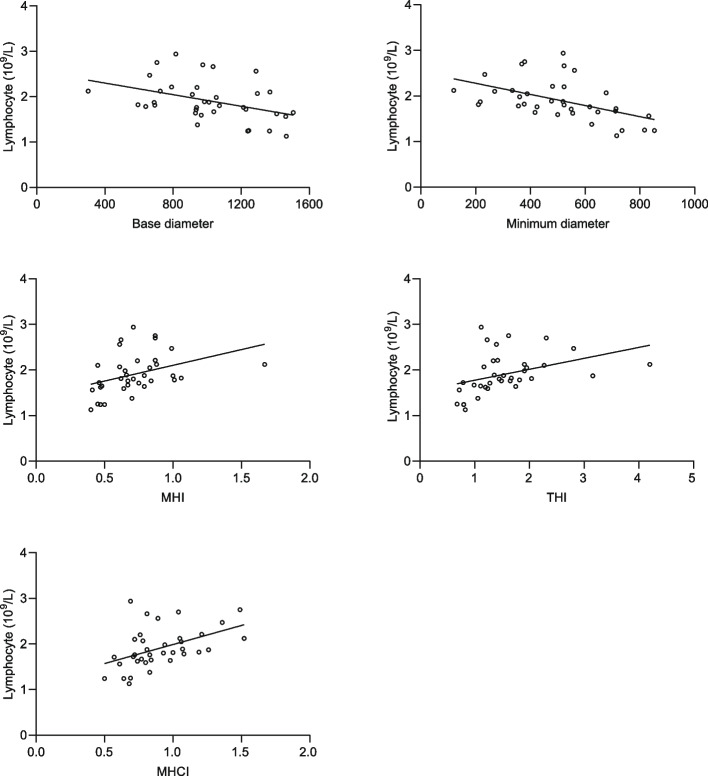
Correlation between lymphocytes and BD, MIN, MHI, THI, MHCI. BD: Base Diameter; MIN: Minimum diameter; MHI: Macular hole index; THI: Tractional hole index; MHCI: Macular hole closure index

In the IMH group, lymphocyte count was negatively correlated with BD and MIN (*p* = 0.013, *p* = 0.002, respectively) and showed a positive correlation with MHI (*p* = 0.002), THI (*p* = 0.000), and MHCI (*p* = 0.001). However, lymphocyte count was not correlated with H (*p* = 0.415), IRC (*p* = 0.108), DHI (*p* = 0.147), and HFF (*p* = 0.079) (shown in Fig. 2, Table 2).

**Table 2:** OCT configuration parameters of IMH and their correlation with lymphocyte counts

Parameters	value	correlation with lymphocyte counts	
		**correlation coefficient**	***p*-value**
**BD (μm)**	1012.35 ± 286.25	-0.411	0.013^a^^,^*
**MIN (μm)**	503.54 ± 184.58	-0.506	0.002^a^^,^*
**H (μm)**	670.41 ± 103.52	0.140	0.415^a^
**IRC (μm**^**2**^**)**	250143.82 ± 135631.90	0.273	0.108^a^
**MHI**	0.72 ± 0.24	0.497	0.002^b^^,^*
**THI**	1.56 ± 0.72	0.568	0.000^b^^,^*
**DHI**	0.50 ± 0.11	-0.247	0.147^a^
**HFF**	0.87 ± 0.22	0.297	0.079^b^
**MHCI**	0.90 ± 0.25	0.543	0.001^b^^,^*

*BD* Base Diameter, *MIN* Minimum diameter, *H* Height, *IRC* Area of the intraretinal cyst, *MHI* Macular hole index, *THI* Tractional hole index, *DHI* Diameter hole index, *HFF* Hole form factor, *MHCI* Macular hole closure index

^*^Statistically significant

^a^Pearson test

^b^Spearman test

**Fig. 3:**
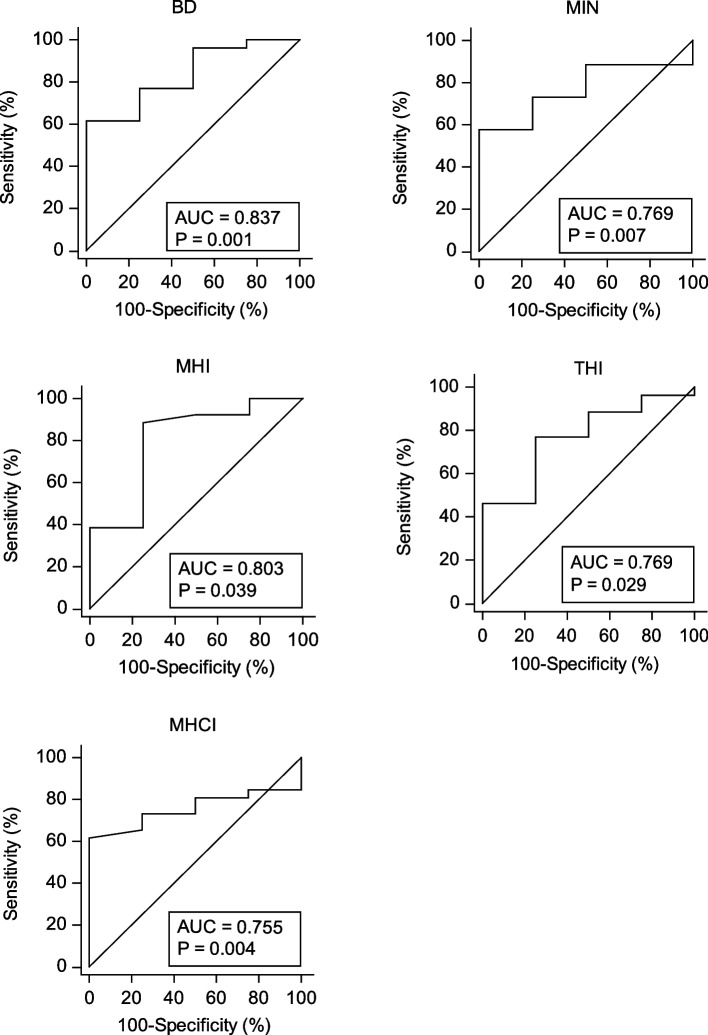
ROC curve analysis of the OCT parameters as prognostic factor for closure type of IMH. BD: Base Diameter; MIN: Minimum diameter; MHI: Macular hole index; THI: Tractional hole index; MHCI: Macular hole closure index

In the IMH group, six patients were lost to follow-up and the remaining 30 patients completed the 1-month follow-up. These 30 patients were divided into type 1 and type 2 closure groups. The ROC curves of the OCT parameters as prognostic factor for closure type of IMH are shown in Fig. 3. BD, MIN, MHI, THI, and MHCI were significant predictors of anatomical outcomes (*p* = 0.001, *p* = 0.007, *p* = 0.039, *p* = 0.029, *p* = 0.004, respectively). However, no significant area under the ROC curve was obtained for H (*p* = 0.695), IRC (*p* = 0.954), DHI (*p* = 0.852), and HFF (*p* = 0.696).

The cut-off point for BD was 984.3 μm, with a sensitivity and specificity of 61.5% and 100%, respectively. The cut-off point for MIN was 499.61 μm, with a sensitivity and specificity of 57.7% and 100%, respectively. The cut-off point for MHI was 0.47, with a sensitivity and specificity of 88.5% and 75%, respectively. The cut-off point for THI was 1.2, and the sensitivity and specificity were 76.9% and 75%, respectively. The cut-off point for MHCI was 0.81, with a sensitivity and specificity of 61.5% and 100%, respectively (shown in Table 3).

**Table 3:** ROC curve analysis of the OCT parameters as prognostic factor for closure type of IMH

Parameters	AUC	Cut-off value	Sensitivity (%)	Specificity (%)	95% CI	*p*-value
**BD**	0.837	≤ 984.3	61.5	100.0	0.657–0.945	0.001*
**MIN**	0.769	≤ 499.61	57.7	100.0	0.580–0.902	0.007*
**H**	0.577	> 593.119	84.6	50.0	0.384–0.754	0.695
**IRC**	0.510	≤ 318487.507	76.9	50.0	0.322–0.696	0.954
**MHI**	0.803	> 0.47	88.5	75.0	0.618–0.925	0.039*
**THI**	0.769	> 1.2	76.9	75.0	0.580–0.902	0.029*
**DHI**	0.524	> 0.58	26.9	100.0	0.335–0.708	0.852
**HFF**	0.577	> 0.82	61.5	75.0	0.384–0.754	0.696
**MHCI**	0.755	> 0.81	61.5	100.0	0.564–0.892	0.004*

*BD* Base Diameter, *MIN* Minimum diameter, *H* Height, *IRC* Area of the intraretinal cyst, *MHI* Macular hole index, *THI* Tractional hole index, *DHI* Diameter hole index, *HFF* Hole form factor, *MHCI* Macular hole closure index

^*^Statistically significant

According to the cut-off points, 30 follow-up patients were divided into the above-cut-off and below-cut-off groups. Lymphocyte counts were compared between the groups. There was no significant difference in BD between the two groups. Compared to the MIN > 499.61 μm group, lymphocyte count was significantly higher in the MIN ≤ 499.61 μm group (*p* = 0.042). Meanwhile, lymphocyte count was significantly higher in the MHI ≥ 0.47 group (*p* = 0.016), THI ≥ 1.2 group (*p* = 0.009), and MHCI ≥ 0.81 group (*p* = 0.024) compared to the below-cut-off groups (shown in Table 4).

**Table 4:** Lymphocyte counts of the above-cut-off and below-cut-off groups

	**Above cut off (10**^**9**^**/L)**	**Below cut off (10**^**9**^**/L)**	***p*-value**
**BD**	1.81 ± 0.46	2.05 ± 0.45	0.151^a^
**MIN**	1.82 ± 0.54	2.06 ± 0.34	0.042^a^^,^*
**MHI**	2.03 ± 0.44	1.56 ± 0.35	0.016^a^^,^*
**THI**	2.06 ± 0.36	1.66 ± 0.56	0.009^a^^,^*
**MHCI**	2.06 ± 0.37	1.78 ± 0.53	0.024^a^^,^*

*BD* Base Diameter, *MIN* Minimum diameter, *MHI* Macular hole index, *THI* Tractional hole index, *MHCI* Macular hole closure index

^*^Statistically significant

^a^Mann-Whitney U test

## Discussion

To the best of our knowledge, this is the first study to demonstrate a close relationship between serum inflammatory markers and IMH. In our study, the lymphocyte counts in the IMH group were significantly higher than those in the control group. There were no significant differences in other data between the two groups.

Our clinical results are consistent with those of preliminary studies. Studies have shown that owing to the anatomical structure of the retina, T lymphocytes can contact Müller cells after passing through the capillary wall. In vitro studies have shown that Müller cells inhibit the proliferation of T helper lymphocytes, and experiments have shown that specific destruction of Müller cells in animals increases the incidence of experimental autoimmune uveitis [28]. Müller cells are damaged during the formation of a MH; afterwards, T helper lymphocytes may proliferate. Once IMH forms, the retina undergoes a process called reactive gliosis [29]. Lymphocytes disrupt the blood-retinal barrier and migrate to the vitreous cavity. There, lymphocytes come into contact with transitional pigment cells and glial cells, secreting cytokines that promote inflammatory responses and enhance macrophage-mediated phagocytosis [30]. Significant differences in cytokine levels in the aqueous humor were found between patients with IMHs and those with cataracts. Significant changes in cytokine levels in patients with IMHs are due to inflammatory responses [4]. In addition, lymphocytes in the vitreous cavity can promote the transformation of glial cells into fibroblasts and accelerate tissue hyperplasia repair [4]. The recruitment of innate immune cells, which leads to the recruitment of T cells and other immune cells, can also induce damage repair [31]. We hypothesized that lymphocytes are not pathogenic factors in IMH, but play an important role in the tissue repair of IMH.

Lymphocytes and neutrophils are important components of leukocytes that mediate adaptive and innate immunity. Neutrophils play an important role in initiating and regulating immune processes [32]. Meanwhile, lymphocytes are specific inflammatory mediators with regulatory and protective effects. Consistent evidence suggests that the infiltration of lymphocytes into solid tumors is a beneficial prognostic marker [33]. Platelets can bind to leukocytes and endothelia and influence the function of inflammatory factors. Blood cell parameters, including lymphocyte counts, neutrophil counts, monocyte counts, platelet count, NLR, MLR, PLR, and SII, serve as simple inflammatory markers in diseases. Once a high inflammatory response occurs, the counts of neutrophils, platelets, and monocyte increase overall, whereas lymphocyte counts decrease overall. However, in our study, there were no significant changes in the counts of neutrophils, monocytes, and platelets, whereas lymphocyte counts increased in the IMH group. We assumed that during the MH formation phase, lymphocyte activation increased the release of related inflammatory factors and promoted MH repair. Inflammation and cytokines may be involved in the formation of MH; however, IMH is not a hyper-inflammatory disease.

With the advent of OCT, which is a useful diagnostic tool for fundus disorders, observation of the progression and prediction of visual outcomes after IMH surgery has become convenient. In our study, we investigated various measurements and indices, including BD, MIN, H, IRC, MHI, THI, DHI, HFF, and MHCI. These parameters were used to assess macular morphology and predict postoperative anatomical outcomes.

We investigated the correlation between lymphocyte counts and these parameters. In the IMH group, we found that lymphocyte count had significantly negative correlations with BD and MIN, but had significantly positive correlations with MHI, THI, and MHCI. Some researchers found that BD and MIN might reflect tangential traction during the formation of IMH and have negative correlations with IMH closure rates [34, 35]. MHI is highly correlated with postoperative anatomical outcomes and is a good predictor of anatomically successful closure of IMH. THI was found to have a significant positive correlation with optimal postoperative correction [36]. When the MHCI is between 0.7 and 1.0, successful closure with normal foveal morphology may be achieved [37]. Our ROC curve analysis showed similar results. Regarding the cut-off points of the ROC curves, lymphocyte count was significantly higher in the group with MIN ≤ 499.61 μm, and it was significantly higher in the MHI ≥ 0.47, THI ≥ 1.2, and MHCI ≥ 0.81 groups. According to previous studies, lymphocytes can accelerate tissue hyperplasia repair [4]. Therefore, we hypothesized that the MH healed better when the MIN was ≤ 499.61 μm, MHI was ≥ 0.47, THI was ≥ 1.2, or MHCI was ≥ 0.81.

This study had some limitations. First, the sample size was relatively small, and the data were obtained from a single center. Second, this was a retrospective study. The complete blood count of the patients used in this study was extracted from only one blood test, which may not accurately predict the persistence of blood parameters over time. Further investigation is needed, especially regarding the blood samples of the postoperative patients. Meanwhile, third, the study lacked additional examinations of blood or eye tissues to test inflammatory markers such as cytokines and laser flare cell values.

## Conclusions

Inflammation might not be an initiating factor, but it plays a relatively important role in IMH formation. Furthermore, lymphocytes might be involved in the developmental and postoperative recovery phases of IMH. The result of the study could provide new insights into the development and progression of IMH. This was a retrospective study, and we used complete blood counts as test specimens; however, the number of clinical cases was insufficient. In addition, the lack of detailed analysis of inflammation was a limitation in our study. In the future, intraocular tissue samples from patients with MHs should be used to further clarify the role of lymphocytes in the pathogenesis of IMH.

## Data Availability

The datasets used and analysed during the current study are available from the corresponding author on reasonable request.

## Abbreviations

BD: Base diameter
DHI: Diameter hole index
H: Height
HFF: Hole form factor
ILM: Internal limiting membrane
IMH: Idiopathic macular hole
IRC: Intraretinal cyst
MH: Macular hole
MHCI: Macualr hole closure index
MHI: Macular hole index
MIN: Minimum
MLR: Monocyte-to-lymphocyte ratio
NLR: Neutrophil-to-lymphocyte ratio
OCT: Optical coherence tomography
PLR: Platelet-to-lymphocyte ratio
ROC: Receiver operating characteristic
SII: Systemic immune-inflammation index
THI: Tractional hole index
